# 

**Published:** 1977

**Authors:** 


					W/
^BR
j
?fi?M
NATION A!.
LIBRARY
5 J UN J979 j
OF *"9
^MEDICINE
KjuJ
EDICO
CHIRURGICAL
JULY/OCTOBER 1977
VOLUME 92 (iii/iv)
NUMBER 343/344

				

